# Three Outbreak-causing *Neisseria meningitidis Serogroup* C Clones, Brazil^1^

**DOI:** 10.3201/eid1911.130610

**Published:** 2013-11

**Authors:** David E. Barroso, Terezinha M.P.P. Castiñeiras, Fernanda S. Freitas, Jane W. Marsh, Mary G. Krauland, Mary M. Tulenko, Érica L. Fonseca, Ana C.P. Vicente, Maria C. Rebelo, Elaine O. Cerqueira, Adriano C. Xavier, Ana P.C.M. Cardozo, Simone E.M. da Silva, Lee H. Harrison

**Affiliations:** Oswaldo Cruz Institute, Oswaldo Cruz Foundation, Rio de Janeiro, Brazil (D.E. Barroso, F.S. Freitas, É.L. da Fonseca, A.C.P. Vicente);; Federal University School of Medicine, Rio de Janeiro (T.M.P.P. Castiñeiras);; University of Pittsburgh, Pittsburgh, Pennsylvania, USA (J.W. Marsh, M.G. Krauland, M.M. Tulenko, L.H. Harrison);; State Department of Health, Rio de Janeiro (M.C. Rebelo, E.O. Cerqueira);; Cientificalab Laboratory Products and Systems, Rio de Janeiro (A.C. Xavier, A.P.C.M. Cardozo, S.E.M. da Silva).

**Keywords:** Neisseria meningitidis, serogroup C, bacteria, clones, epidemiology, public health, population surveillance, data collection, Brazil

## Abstract

During 2003–2012, 8 clusters of meningococcal disease were identified in Rio de Janeiro State, Brazil, all caused by serogroup C *Neisseria meningitidis*. The isolates were assigned to 3 clonal complexes (cc): cc11, cc32, and cc103. These hyperinvasive disease lineages were associated with endemic disease, outbreaks, and high case-fatality rates.

The last epidemic of *Neisseria meningitidis* serogroup C meningococcal disease in Rio de Janeiro State, Brazil, occurred in 1994. It was caused by C:2b:P1.10 isolates that belonged to cluster A4 (*1*). Although the number of cases of serogroup C disease subsequently declined after a vaccination campaign, rates of serogroup C disease again began to increase in 2000. During 2003–2012, public health surveillance identified 8 clusters of serogroup C meningococcal disease in Rio de Janeiro State. We report the investigation of these meningococcal disease clusters and typing information of the causative agent.

## The Study

Public health surveillance of meningococcal disease in Rio de Janeiro State is conducted by the Meningitis Advisory Committee of the State Department of Health, which uses data obtained from 2 surveillance sources: mandatory reports of meningococcal disease cases and reports of laboratory-confirmed *N. meningitidis* isolates collected by the Central Laboratory Noel Nutels and the Infectious Diseases State Institute São Sebastião, which are state reference laboratories, and 1 outsourced laboratory for bacterial meningitis (Cientificalab Laboratory Products and Systems, Rio de Janeiro, Brazil). Chemoprophylaxis with rifampin is currently recommended for close contacts of persons with confirmed or suspected cases of meningococcal disease.

A cluster was defined as ≥3 cases of meningococcal disease with a clear epidemiologic link and with *N. meningitidis* of the same serogroup recovered from either a normally sterile site or detected by PCR. Reports of invasive meningococcal disease during 2000–2012 were obtained from the Meningitis Advisory Committee and analyzed by using EpiInfo (version 3.5.3; Centers for Disease Control and Prevention, Atlanta, GA, USA). This study was approved by the Ethical Committee of the Evandro Chagas Research Institute of the Oswaldo Cruz Foundation.

We identified 8 clusters involving 46 cases that occurred during 2003–2012; all were caused by serogroup C *N. meningitidis* (Technical Appendix, Figure). *N. meningitidis* serogroup was determined by slide agglutination with specific rabbit antisera (BD Difco, Sparks, MD, USA) or serogroup-specific PCR directly from cerebrospinal fluid samples (*2*). Serotype and serosubtype were determined by immunoblot analysis at the National Meningitis Reference Center. Susceptibility to rifampin was determined by using E-test (bioMérieux, Marcy-l'Étoile, France).

**Figure:**
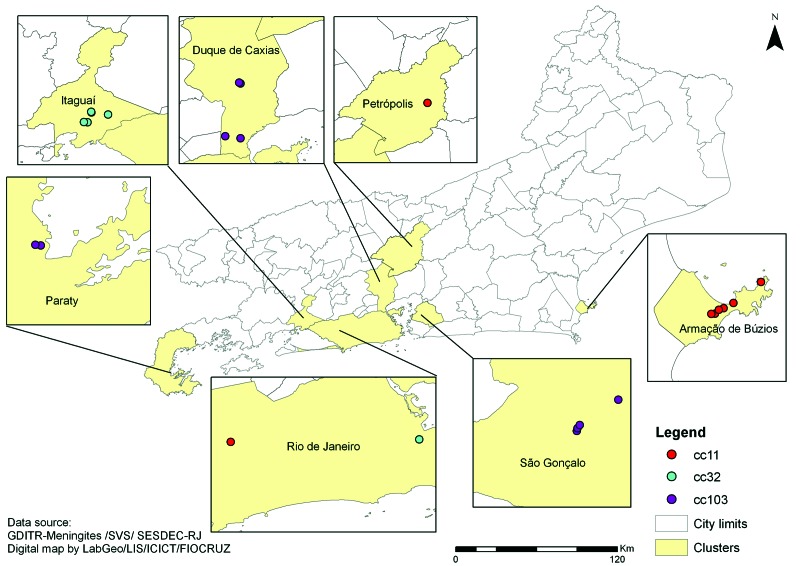
Spatial distribution of 8 meningococcal disease clusters caused by 3 different clonal complexes (cc) of *Neisseria meningitidis*, Rio de Janeiro State, Brazil, 2003–2012.

The genetic lineage of *N. meningitidis* isolates recovered in culture (n = 11) or directly detected in cerebrospinal fluid samples (n = 24) was determined by multilocus sequence typing (MLST), and the antigenic profile was determined by sequencing antigen-encoding genes: *porB*, *porA* (variable regions 1 and 2), and *fetA* variable region (*3*). A total of 122 serogroup C invasive isolates (C:2a [22]; C:2b [17]; C:4,7 [36]; C:19 [4]; C:23 [43]) recovered from 1990 through 2010 were also genotyped. Sequence types and alleles at antigenic loci were assigned by the *N. meningitidis* MLST database (www.mlst.net).

Serogroup C disease increased from 121 (26%) of 463 cases during 2000–2003, to 174 (44%) of 394 cases during 2004–2007, and to 499 (84%) of 594 cases during 2008–2012 (p<0.01). The case-fatality rate of serogroup C disease also increased during the same periods: 12%, 14%, and 19% (p = 0.03), respectively. These serogroup C isolates were mainly represented by 4 serologic phenotypes: C:23:P1.14–6 (60%), C:4,7:P1.7,1 (15%), C2a:P1.5,2 (12%), and C:4,7:P1.19,15 (6%).

New cases associated with serogroup C meningococcal disease clusters occurred within an average of 15 days (range 1–30 days), and those infected had proximity to each other (same household, vicinity, daycare center, primary school, or workplace). The average age of patients was 13 years (range 10 months–51 years). The clinical signs and symptoms recorded when the person sought medical treatment were fever (98%), vomiting (80%), hemorrhagic rash (67%), headache (65%), neck stiffness (50%), impaired consciousness (41%), diarrhea (15%), abdominal pain (15%), convulsions (13%), sore throat (9%), and myalgia (6%). The overall case-fatality rate was 28% (13/46), ranging from 17% (cc103) to 44% (cc11) (Table, Appendix); 6 (46%) of 13 deaths occurred as the person sought treatment. A program of vaccination with serogroup C polysaccharide vaccine was implemented twice, once in October 2003 (Paraty) and again in January 2008 (Armação de Búzios). Subsequently, 1 vaccinated person became infected in each locality (Technical Appendix).

**Table T1:** Genotyping of serogroup C *Neisseria meningitidis* invasive isolates recovered from 1990 to 2010 with timeline showing when the cluster-associated clone were first observed in Rio de Janeiro State, Brazil

Serogroup/year	Genotype (no. isolates)/clonal complex	Date of emergence of cluster-related clone (date of commencement of cluster)
C:2a	ST-11	
1990	2-184:P1.5-1,2-2:F1-1:ST-5121 (1)	
1995–1997	2-2:P1.5,2:F3-6:ST-11 (5)	
1997	2-60:P1.5,2:F3-6:ST-7849 (1)	
2000	2-145:P1.5,2:F1-1:ST-11 (1)	
2001	2-184:P1.5-1,2-2:F1-1:ST-5121 (1)	
2007–2009	2-2:P1.5-1,10-8:F3-6:ST-11 (9)	February 2007 (February 2007, January 2008)
2009	2-2:P1.5-1,5-11:F3-6:ST-11 (1)	
2010	2-2:P1.5-1,10-8:F3-6:ST-9452 (3)	May 2010 (May 2010)
C:2b	ST-8	
1990	2-3:P1.18-1,3:F3-1:ST-8 (1)	No cluster associated with this clonal complex
1992	2-3:P1.18-1,3:F3-9:ST-8 (2)	
1995–1997	2-30:P1.5-2,10:F5-2:ST-8 (1)	
	2-3:P1.18-1,3:F3-1:ST-8 (1)	
	2-30:5-2,10:F5-2:ST-153 (8)*	
	2-3:18-1,3:F3-9:ST-7769 (1)	
	2-30:5-2,10:F5-2:ST-7713 (1)	
2002^†^	2-30:5-2,10:F5-2:ST-153 (1)	
	2-30:5-2,10:F5-2:ST-7705 (1)	
C:4,7	ST-32	
1994	3-1:P1.19,15:F5-1:ST-7709 (1)	
1998–2010	3-79:P1.7-1,1:F5-1:ST-639 (16)	June 1998 (July 2009)
	3-1:P1.19,15:F1-80:ST-33 (1)	
	3-299:P1.7-1,1:F5-1:ST-639 (2)	
	3-1:P1.19,15:F5-1:ST-33 (2)	
	3-79:P1.19,15:F5-1:ST-639 (1)	
	3-1:P1.19,15:F5-1:ST-34 (1)	
	3-1:P1.19,15:F5-1:ST-639 (3)	
	3-79:P1.7-1,1:F5-1:ST-7692 (1)	
	3-79:P1.7-1,1:F5-1:ST-7696 (1)	October 2006 (October 2006)
	3-294:P1.7-1,1:F5-1:ST-639 (1)	
2007	3-300:P1.18-1,3:F5-37:ST-41 (1)	No cluster associated with this clonal complex
C:4,7	No clonal complex	
1993–1994	3-27:P1.20,9:F1-7:ST-7690 (3)	No cluster associated with these strains
	3-1:P1.21,16:F1-20:ST-7712 (1)	
2003	3-1:P1.5,15:F4-3:ST-7691 (1)	
C:19	ST-41/44, ST-174, and ST-269	
1993–1995	3-295:P1.5-1,10-4:F4-21:ST-3772 (1)	No cluster associated with these clonal complexes
	3-295:P1.5-1,10-4:F5-1:ST-3772 (1)		
2007	3-35:P1.21,16:F5-13:ST-7817 (1)		
	3-71:P1.19,15:F5-2:ST-437 (1)		
C:23	ST-103		
2001–2010	2-23:P1.22,14-6:F1-80:ST-3779 (1)		
	2-23:P1.22,14-6:F3-9:ST-3779 (13)	July 2001 (February 2012)	
	2-23:P1.22,14-6:F5-92:ST-7689 (1)		
	2-23:P1.22,14-6:F3-9:ST-3780 (14)	July 2003 (April 2012)	
	2-23:P1.22,14-6:F1-5:ST-5727 (1)		
	2-23:P1.22,14-6:F3-9:ST-7708 (1)	September 2003 (September 2003)	
	2-23:P1.5,14-6:F3-9:ST-5122 (1)		
	2-23:P1.22,14-6:F3-9:ST-5122 (2)		
	2-23:P1.22,14-6:F5-92:ST-5338 (1)		
	2-167:P1.22,14-6:F3-9:ST-3779 (3)		
	2-23:P1.18-1,3:F3-9:ST-3779 (3)		
	2-23:P1.22,14-6:F3-9:ST-8732 (1)		
C:23	No clonal complex		
2010	2-23:P1.22,14-6:F3-9:ST-8730 (1)	No cluster associated with this strain

*No invasive C:2b isolate has been recovered since 2002. †The clone associated with the last epidemic of serogroup C disease in 1994.

Isolates assigned to clonal complex (cc) 11, cc32, and cc103 were associated with the clusters of meningococcal disease (Technical[Table T1] Appendix; Figure); all were rifampin-susceptible (MICs, 0.006–0.19 μg/mL). The results of genotyping the 122 invasive isolates collected from 1990 through 2010 are shown in the Table. The Table also indicates when the cluster-associated clones were first observed.

## Conclusions

Clusters of meningococcal disease were a prominent feature of *N. meningitidis* infections in several countries during the 1990s (*4*,*5*). These meningococcal clusters have been associated with educational institutions and particular clones of serogroup C. Clusters and community outbreaks of serogroup C disease have recently been observed in Brazil with increasing frequency outside the person’s place of residence and involving teenagers and young adults, e.g., caused by the ST-3780 (cc103) isolates (*6*–*8*). A single cluster has been associated with the C:4,7:P1.19,15 phenotype (*9*).

Although the annual incidence rate remained stable (2–3 cases/100,000 population), clusters of meningococcal disease marked a change in the epidemiology of *N. meningitidis* infection during the 2000s in Rio de Janeiro State, while serogroup C disease and its case-fatality rate steadily increased. These clusters were caused by different clones, involved mostly children, and were accompanied by high case-fatality rates. The serogroup C clones found in this study seem to have emerged during the 2000s and are also now the major cause of endemic meningococcal disease. Some of these clones, namely, cc11 and cc32, have undergone capsular switching. For instance, the 2–2:P1.5–1,10–8:F3–6:ST-7816 (a single locus variant of ST-11) clone from 2009 was found to express a serogroup W capsule (*10*), and the 3–79:P1.7–1,1:F5–1:ST-639 clone was previously demonstrated to belong to serogroup B (*3*).

Chemoprophylaxis to control clusters has been ineffective in preventing new cases, possibly because transmission might have been occurring among social networks that did not receive chemoprophylaxis. In addition, it is not known whether chemoprophylaxis reduces risk in educational institutions (*5*). All of these clusters were potentially vaccine preventable with monovalent serogroup C meningococcal vaccine, which was instituted in the state program of routine vaccination for children (<2 years) in October 2010. The implementation of molecular surveillance is advisable to both guide immunization programs and to monitor the effects of the immunization program and its consequences for qhe population biology of *N. meningitidis* associated with invasive disease.

Technical Appendix Table showing characteristics of patients from clusters of serogroup C meningococcal disease, Rio de Janeiro State, Brazil, 2003–2012
