# Supplementary material for: Three Outbreak-causing Neisseria meningitidis Serogroup C Clones, Brazil
Source: Emerg Infect Dis. 2013 Nov;19(11):1847–50. doi: 10.3201/eid1911.130610 (PMC3837672; doi:10.3201/eid1911.130610)
Supplement: Technical Appendix — Table showing characteristics of patients from clusters of serogroup C meningococcal disease, Rio de Janeiro State, Brazil, 2003–2012 [file 13-0610-Techapp-s1.pdf]

Article DOI: http://dx.doi.org/10.3201/eid1911.130610

Three Outbreak-causing *Neisseria meningitidis Serogroup* C Clones, Brazil

Technical Appendix

Technical Appendix Table. Description of patients from clusters of serogroup C meningococcal disease, Rio de Janeiro State, Brazil, 2003–2012
ST-11 clonal complex (C:2a:P1.5)
Overall case-fatality rate = 44%; 63% of the patients with septicemia.

Locality (year), no. cases	Date*	Setting or Profession	Age, y	Clinical manifestation	Outcome	Genotype	Annual incidence rate (population)
Petrópolis (2007)							7.5/100,000 (295,000)
1	02/20	Daycare	2	Septicemia	Deceased	2-2:P1.5-1,10-8:F3-6:ST-11 (cc11)	
2	02/20	Daycare	3	Meningitis + septicemia	Discharged	2-2:P1.5-1,10-8:F3-6:ST-11 (cc11)	
3	02/20	Daycare	3	Septicemia	Discharged	Not available	
4	02/21	Daycare	3	Septicemia	Discharged	2-2:P1.5-1,10-8:F3-6:ST-11 (cc11)	
5	02/21	Daycare	2	Septicemia	Discharged	Not available	
6	02/26	Daycare	1	Septicemia	Discharged	2-2:P1.5-1,10-8:F3-6:ST-11 (cc11)	
7	02/27	Daycare	19	Meningitis	Deceased	2-2:P1.5-1,10-8:F3-6:ST-11 (cc11)	
Armação de Búzios (2008)							22/100,000 (27,000)
1	01/06	Waiter	25	Septicemia	Discharge	Not available	
2	01/12	Street vendor	14	Meningitis + septicemia	Deceased	2-2:P1.5-1,10-8:F3-6:ST-11 (cc11)	
3	01/12	Housemaid	18	Meningitis	Discharge	2-2:P1.5-1,10-8:F3-6:ST-11 (cc11)	
4	01/13	Tourist	46	Meningitis + septicemia	Discharge	2-2:P1.5-1,10-8:F3-6:ST-11 (cc11)	
5	01/13	Street vendor	18	Septicemia	Deceased	2-2:P1.5-1,10-8:F3-6:ST-11 (cc11)	
6	09/09	Laborer (vaccine failure)	51	Meningitis	Discharge	2-2:P1.5-1,10-8:F3-6:ST-11 (cc11)	
Rio de Janeiro (2010)							3/100,000 (6,300,000)
1	05/03	Household	2	Septicemia	Deceased	2-2:P1.5-1,10-8:F3-6:ST-9452 (cc11)	
2	05/03	Household	2	Septicemia	Deceased	2-2:P1.5-1,10-8:F3-6:ST-9452 (cc11)	
3	05/21	Household	17	Septicemia	Deceased	2-2:P1.5-1,10-8:F3-6:ST-9452 (cc11)	

ST-32 clonal complex (C:4,7:P1.7,1)
Overall case-fatality rate = 25%; 25% of the patients with septicemia.

Locality (year), no. cases	Date*	Setting or Profession	Age, y	Clinical manifestation	Outcome	Genotype	Annual incidence rate (population)
Rio de Janeiro (2006)							2.7/100,000 (6,000,000)
1	10/19	Workplace	30	Septicemia	Discharge	3-79:P1.7-1,1:F5-1:ST-7696 (cc32)	
2	10/23	Workplace	20	Septicemia	Discharge	Not available	
3	10/24	Workplace	17	Meningitis + septicemia	Discharge	3-79:P1.7-1,1:F5-1:ST-7696 (cc32)	
4	10/24	Workplace	19	Meningitis	Discharge†	3-79:P1.7-1,1:F5-1:ST-7696 (cc32)	
Itaguaí (2009)							12/100,000 (109,000)
1	02/24	Vicinity	3	Meningitis	Discharge	3-79:P1.7-1,1:F5-1:ST-639 (cc32)	
2	07/23	Household	7	Meningitis	Deceased	Not available	
3	07/23	Household	6	Meningitis + septicemia	Discharge	3-79:P1.7-1,1:F5-1:ST-639 (cc32)	
4	08/04	Vicinity	5	Septicemia	Deceased	Not available	
5	08/04	Household	2	Meningitis + septicemia	Discharge	Not available	
6	08/05	Household	12	Meningitis + septicemia	Discharge	3-79:P1.7-1,1:F5-1:ST-639 (cc32)	
7	08/10	Vicinity	34	Meningitis	Deceased	3-79:P1.7-1,1:F5-1:ST-639 (cc32)	
8	08/12	Household	10	Meningitis	Discharge	Not available	

ST-103 clonal complex (C:23:P1.14-6)
Overall case-fatality rate = 17%; 11% of the patients with septicemia.

Locality (year), no. cases	Date*	Setting or Profession	Age, y	Clinical manifestation	Outcome	Genotype	Annual incidence rate (population)
Paraty (2003)							
1	09/04	Primary school	9	Meningitis + septicemia	Discharge	2-23:P1.22,14-6:F3-9:ST-7708 (cc103)	10.2/100,000 (37,000)
2	09/21	Primary school	7	Meningitis	Deceased	Not available	
3	10/12	Primary school	12	Meningitis + septicemia	Discharge‡	Not available	
Paraty (2004)							13.6/100,000 (37,000)
1	01/17	Primary school (vaccine failure)	10	Meningitis + septicemia	Discharge	2-23:P1.22,14-6:F3-9:ST-7708 (cc103)	
2	01/18	Vicinity	42	Meningitis + septicemia	Discharge	2-23:P1.22,14-6:F3-9:ST-3780 (cc103)	
3	03/12	Vicinity	25	Meningitis + septicemia	Discharge	Not available	
4	03/16	Vicinity	32	Meningitis	Discharge	2-23:P1.22,14-6:F3-9:ST-3780 (cc103)	
Duque de Caxias (2012)							3.5/100,000 (855,000)
1	02/28	Primary school	6	Meningitis + septicemia	Discharge	2-23:P1.22,14-6:F3-9:ST-3779 (cc103)	
2	02/28	Primary school	8	Septicemia	Discharge	2-23:P1.22,14-6:F3-9:ST-3779 (cc103)	
3	02/29	Primary school	5	Meningitis +	Discharge	2-23:P1.22,14-6:F3-9:ST-3779	

Locality (year), no. cases	Date*	Setting or Profession	Age, y	Clinical manifestation	Outcome	Genotype	Annual incidence rate (population)
				septicemia		(cc103)	
4	05/14	Vicinity	5	Septicemia	Deceased	2-23:P1.22,14-6:F3-9:ST-3779 (cc103)	
5	05/15	Vicinity	18	Meningitis + septicemia	Deceased	2-23:P1.22,14-6:F3-9:ST-3779 (cc103)	
São Gonçalo (2012)							3/100,000 (999,000)
1	04/19	Vicinity	6	Meningitis	Discharge	2-23:P1.22,14-6:F3-9:ST-3780 (cc103)	
2	05/09	Household	5	Meningitis	Discharge	2-23:P1.22,14-6:F3-9:ST-3780 (cc103)	
3	05/16	Household	3	Meningitis	Discharge	2-23:P1.22,14-6:F3-9:ST-3780 (cc103)	
4	05/16	Household	10 mo	Meningitis	Discharge	2-23:P1.22,14-6:F3-9:ST-3780 (cc103)	
5	05/17	Household	1	Meningitis	Discharge	2-23:P1.22,14-6:F3-9:ST-3780 (cc103)	
6	05/17	Household	2	Meningitis + septicemia	Discharge	2-23:P1.22,14-6:F3-9:ST-3780 (cc103)	

*Date of admission.
†Neurological impairment.
‡Hearing loss.
